# Supplementary material for: Informing disaster-risk management policies for education infrastructure using scenario-based recovery analyses
Source: Nat Commun. 2024 Jan 5;15:325. doi: 10.1038/s41467-023-42407-y (PMC10770163; doi:10.1038/s41467-023-42407-y)
Supplement: Supplementary file 1 — Supplementary Information [file 41467_2023_42407_MOESM1_ESM.pdf]

Supplementary Notes

The supplementary notes provide supplementary tables for the analyses presented in the study.

The performance metrics for two of the four criteria (availability of temporary structures and age group of students) used in the multicriteria decision-making analyses are presented in Supplementary Tables 1 and 2.

Supplementary Table 1 – Performance metrics for the availability of temporary structures (Metric based on the assumption that the average lifespan of the temporary structure is four-five years)

		Proportion of buildings in the school that are TLCs [%]				
		80-100	60-80	40-60	20-40	< 20
Age of TLCs at expected completion date of new buildings [years]	< 1	0.25	0.2	0.15	0.1	0.05
	1 – 3	0.5	0.4	0.3	0.2	0.1
	3 – 4	0.75	0.6	0.45	0.3	0.15
	≥ 4	1.0	0.8	0.6	0.4	0.2

Supplementary Table 2 – Performance metric for age group of students

School level	Assumed priority	Metric
Elementary	High	1
Junior high school	Medium	0.5
Senior high school	Low	0

Supplementary Table 3 presents a range of recovery time mitigation and amplification factors based on a survey[1] of observations, interviews, and focus group discussions from published studies that have compared pre- and post-disaster reconstruction projects in lower-middle income countries.

Supplementary Table 3 – Time amplification and mitigation factors for recovery time modeling. Each range of factors has been developed through a literature survey[1] of observations, interviews and focus group discussions carried out by other authors who have compared pre- and post-disaster reconstruction projects in low and lower-middle income countries.

Parameter	Factor
Time amplification factors	
Land dispute resolution	1.25 – 2
Pandemic	1.5 – 3
Delay in material procurement	1.2 – 2.5
Hostile political conditions	1.5 – 5
Poor management skills	1.5 – 3
Funds disbursement	1.25 – 3
Technical delays	1.2 – 2.5
Time mitigation factor	
Community participation	0.5 – 0.9

References

1. Opabola, E. A., and Galasso, C. "A Probabilistic Framework for Post-Disaster Recovery Modeling of Buildings and Electric Power Networks in Developing Countries," *Reliability Engineering & System Safety*, 2023, p. 109679.
